# Genetic mechanisms of dynamic functional connectivity density in diabetic retinopathy brains: a combined transcriptomic and resting-state functional magnetic resonance imaging study

**DOI:** 10.3389/fncel.2025.1476038

**Published:** 2025-04-10

**Authors:** Yu-Lin Zhong, Hao Liu, Xin Huang

**Affiliations:** ^1^Department of Ophthalmology, Jiangxi Provincial People’s Hospital, The First Affiliated Hospital of Nanchang Medical College, Nanchang, Jiangxi, China; ^2^School of Ophthalmology and Optometry, Jiangxi Medical College, Nanchang University, Nanchang, Jiangxi, China

**Keywords:** diabetic retinopathy, dynamic functional connectivity density, allen human brain atlas, gene expression, rs-fMRI

## Abstract

**Background:**

Diabetic retinopathy (DR) is a condition characterized by fundus lesions resulting from retinal microvascular leakage and obstruction linked to chronic progressive diabetes mellitus. Previous neuroimaging research has revealed both structural and functional changes in the brains of DR patients. Nevertheless, the variations in dynamic functional connectivity density (dFCD) within the brains of DR patients, along with the underlying molecular mechanisms connected to these changes, have yet to be fully understood.

**Methods:**

Forty-seven diabetic retinopathy (DR) patients and 46 healthy controls (HCs) matched for sex, age, and education were recruited for this study from the Department of Ophthalmology at the Jiangxi Provincial People’s Hospital. All subjects underwent resting-state functional magnetic resonance imaging scans to analyze the differences in dFCD between the two groups. Utilizing the Allen Human Brain Atlas, we conducted spatial correlation analyses integrating transcriptomic and neuroimaging data to pinpoint genes showing correlated expression levels with dFCD alterations in DR patients. Subsequently, we carried out gene enrichment, specific expression, and protein-protein interaction analyses.

**Results:**

In comparison to the HC group, the DR group exhibited significantly reduced dFCD variability in the left anterior cingulum, left superior occipital gyrus, and right postcentral gyrus. The abnormal dFCD variability is linked to 1,318 positively and 1,318 negatively associated genes, primarily enriched for biological functions such as ion channels, synapses, and cellular junctions. Specific expression analysis revealed that these genes were distinctly expressed in Purkinje neurons, cortex, and striatum brain regions. Furthermore, protein-protein interaction (PPI) analyses indicated that these positive and negative genes could organize PPI networks with the support of respective hub genes.

**Conclusion:**

our study identified altered dFCD variability in brain regions linked to visual and cognitive functions in DR patients. Moreover, transcriptome-neuroimaging correlation analyses revealed a spatial association between these dFCD changes and the genes with unique functional profiles. These genes were enriched in biologically significant functions and pathways, specific to certain cells and brain areas. Our research offers novel understandings of the genetic mechanisms influencing dFCD alterations in DR.

## Introduction

Diabetes mellitus (DM) is a prevalent metabolic disorder associated with various microvascular complications (Xu, 2015; Bahtiyar et al., 2016; Bansal et al., 2023), among these, DR is a significant microvascular complication affecting the retina in diabetic individuals and stands as a leading cause of global blindness (Wong et al., 2016). A survey conducted in 2020 revealed a worldwide DR prevalence of 22.27% in diabetic populations, impacting approximately 103.12 million individuals (Teo et al., 2021). Risk factors for DR include hypertension, dyslipidemia, obesity, pregnancy, and prolonged diabetes duration (Lopes de Faria et al., 2011; Ting et al., 2016; Wondmeneh and Mohammed, 2024). The primary pathological manifestations of DR encompass capillary non-perfusion, vascular leakage, and degeneration, progressing to proliferative retinal detachment and eventual vision loss. Notably, retinal vessels share similarities in anatomy, physiology, and embryology with cerebral vasculature (Kwa et al., 2002; van de Kreeke et al., 2018). Notably, retinal vessels share similarities in anatomy, physiology, and embryology with cerebral vasculature (Hägg et al., 2013; Sanahuja et al., 2016). Individuals with DR face heightened susceptibility to dementia and Alzheimer’s disease (Ciudin et al., 2017; Biessels and Despa, 2018), and potential cognitive impairment (Crosby-Nwaobi et al., 2013; Chai et al., 2024). While evidence indicates brain alterations and abnormal central nervous system (CNS) activity in DR patients, the underlying neurophysiological mechanisms necessitate further exploration.

Resting-state functional magnetic resonance imaging (rs-fMRI), a non-invasive imaging technique, enables the examination of spontaneous intrinsic brain activity by measuring fluctuations in blood oxygen level-dependent (BOLD) signals without specific tasks or stimuli (Fox and Raichle, 2007; Barkhof et al., 2014). Studies using resting-state functional MRI have revealed diverse patterns of spontaneous brain activity alterations in DR patients, including amplitude of low-frequency fluctuations (ALFF), dynamic ALFF (dALFF), regional homogeneity (ReHo), and functional network connectivity (FNC), predominantly observed in brain regions like the middle occipital gyrus, calcarine, anterior cingulate gyrus, posterior cerebellar lobe, and networks associated with visual and cognitive functions (e.g., visual network, default mode network, executive control network, and salience network). These findings shed light on the neural mechanisms underlying visual and central nervous system abnormalities in DR (Huang et al., 2019, 2020, 2021a; Liao X.-L et al., 2019; Qi et al., 2020; Shi et al., 2022). Functional connectivity density (FCD) analysis, an extension of the FC method, assesses the importance of network nodes in functional integration by measuring their total connectivity strength with all other nodes in the brain network (Lv et al., 2018; Tomasi and Volkow, 2010). This approach, without requiring prior assumptions, is suited for exploratory analyses (Tomasi et al., 2016; Pang et al., 2017; Zhang et al., 2017). Moreover, the human brain is a complex dynamic system with non-stationary neural activity and constantly changing neural interactions (Hutchison et al., 2013; Calhoun et al., 2014; Liao W. et al., 2019), implying the importance of investigating dynamic neural communication across different brain regions in DR patients utilizing the dFCD approach. However, the specific alterations in dFCD among DR patients remain unclear.

DR has a hereditary component, with estimated heritability ranging from 6 to 33% (Kim et al., 2022). Multiple studies have demonstrated familial clustering of DR (Zhang et al., 2010; Cho and Sobrin, 2014), with notably high concordance rates among family members; 65% in twins with type 1 diabetes and 95% in twins with type 2 diabetes (Leslie and Pyke, 1982). These findings imply the genetic regulation of DR onset and severity. Genome-wide association studies (GWAS) have the potential to identify genetic risk loci associated with DR. A comprehensive meta-analysis has revealed several gene loci linked to DR that are crucial for cell survival, insulin signaling, and angiogenesis (Grassi et al., 2011). Moreover, various studies have highlighted the influence of genetic factors on resting-state brain function (Fornito et al., 2011). Nevertheless, the genetic underpinnings of neuroimaging changes in DR patients are not effectively captured by GWAS. This challenge is compounded by the fact that most loci identified by GWAS are situated in regulatory, rather than coding, regions of the genome, posing significant challenges for their biological interpretation (Edwards et al., 2013; Tam et al., 2019). Spatial correlation analyses integrating transcriptomics and neuroimaging data from the Allen Human Brain Atlas (AHBA), known for its case-control imaging discrepancies, were conducted to explore the genetic basis of macroscopic neuroimaging alterations (Arnatkeviciute et al., 2022; Xue et al., 2022). This method is widely used to unveil gene expression patterns associated with structural and functional variations in patients’ brains, although investigations on the genetic mechanisms underlying dFCD changes in DR are lacking.

Therefore, In our study, we initially assessed and compared dFCD differences between DR patients and controls. Subsequently, utilizing gene expression data from the AHBA database integrated with dFCD maps, we conducted spatial correlation analyses to identify genes whose expression levels correlated with dFCD changes in DR. Finally, gene enrichment, specific expression, and protein-protein interaction analyses were carried out on the identified genes to elucidate their functions and understand the molecular mechanisms behind dFCD alterations in DR.

## Materials and methods

### Participants

Forty-seven diabetic retinopathy (DR) patients and 46 healthy controls (HCs) matched for sex, age, and education were recruited for this study from the Department of Ophthalmology at the Jiangxi Provincial People’s Hospital.

The inclusion criteria for diabetic retinopathy (DR) were as follows: (1) Fasting blood glucose levels of equal to or greater than 7.0 mmol/L, random blood glucose levels of equal to or greater than 11.1 mmol/L, or 2-h glucose levels of equal to or greater than 11.1 mmol/L; (2) All DR patients were diagnosed with non-proliferative DR without macular edema. The DR classification followed the Early Treatment Diabetic Retinopathy Study grading scheme, encompassing mild and moderate non-proliferative DR, severe non-proliferative retinopathy, and non-high-risk and high-risk proliferative DR.

The exclusion criteria for diabetic retinopathy (DR) were as follows: (1) Patients with proliferative DR complicated by retinal detachment and vitreous hemorrhage; (2) Presence of additional ocular complications (such as cataracts, glaucoma, high myopia, or optic neuropathy); (3) Patients diagnosed with diabetic nephropathy (urine albumin/creatinine ratio > 30 mg/g persisting for more than 3 months); (4) Diabetic neuropathy.

The inclusion and exclusion criteria for healthy controls (HC) were as follows: (1) Fasting blood glucose levels < 7.0 mmol/L, random blood glucose levels < 11.1 mmol/L, glycosylated hemoglobin levels < 6.5%; (2) Absence of ocular diseases (such as myopia, cataracts, glaucoma, optic neuritis, or retinal degeneration); (3) Binocular visual acuity ≥ 1.0; (4) No history of ocular surgery; (5) Absence of psychiatric disorders. All participants met the eligibility criteria for MRI scans.

The study protocol adhered to the Declaration of Helsinki and received approval from the Research Ethics Committee of Jiangxi Provincial People’s Hospital. Before participating in the study, all participants were informed about the study’s objectives, methods, and potential risks, and provided written informed consent to participate.

### MRI acquisition

Functional images were acquired using a gradient-echo-planar imaging sequence. Participants were instructed to maintain a relaxed state with closed eyes, refrain from focusing on specific thoughts, and avoid falling asleep. Whole-brain T1-weighted images were obtained through a three-dimensional brain volume imaging (3D-BRAVO) MRI protocol with the following parameters: repetition time (TR)/echo time (TE) = 8.5/3.3 ms, slice thickness = 1.0 mm, no intersection gap, acquisition matrix = 256 × 256, field of view = 240 × 240 mm^2^, and flip angle = 12°. Functional images were acquired using a gradient-echo-planar imaging sequence with the following parameters: TR/TE = 2,000 ms/25 ms, slice thickness = 3.0 mm, gap = 1.2 mm, acquisition matrix = 64 × 64, flip angle = 90°, field of view = 240 × 240 mm^2^, voxel size = 3.6 × 3.6 × 3.6 mm^3^, and 35 axial slices. All participants were instructed to relax with closed eyes, avoiding specific thoughts or falling asleep.

### Data preprocessing

The preprocessing steps utilized the Data Processing and Analysis of Brain Imaging (DPABI) toolbox (DPABI)^1^ (Yan et al., 2016), a tool based on Statistical Parametric Mapping (SPM12) incorporated in MATLAB 2013a (MathWorks, Natick, MA, United States). The pre-processing protocol consisted of the following steps: (1) Removal of the initial 10 volumes, (2) Correction for slice timing effects and motion, excluding participants with head motion exceeding 2 mm or rotation exceeding 1.5° during scanning (Van Dijk et al., 2012). (3) Normalization of data to Montreal Neurological Institute (MNI) 152 space at a resolution of 3 mm × 3 mm × 3 mm, and (4) Spatial smoothing through convolution with a 6 mm × 6 mm × 6 mm isotropic Gaussian kernel at full width at half maximum.

### Dynamic functional connectivity density analysis

The analysis of dFCD was processed with the DynamicBC software. dFCD analysis was conducted using the sliding-window approach, with a window size of 50 TR and a sliding step of 10 TR. We obtained a global FCD map in each window by computing Pearson’s correlations between the truncated time course of all pairs of voxels within the automated anatomical labeling-90 (AAL-90) atlas (comprising 45 cortical and subcortical regions in each hemisphere) (Tzourio-Mazoyer et al., 2002), yielding a set of sliding-window FCD maps for each subject. We used *r* = 0.2 as the correlation coefficient threshold to define the connectivity between two voxels. If their correlation coefficient was larger than 0.2, then connectivity was present between them. The threshold was selected to eliminate the weak correlations induced by noise (Li et al., 2019). Subsequently, the temporal variability was estimated by computing the SD of FCD across sliding windows. In consideration that the global signal regression may induce controversial negative correlations (Fox et al., 2009; Murphy et al., 2009), all our analyses were performed based on positive correlation above a threshold of 0.2.

### Brain gene expression data processing

Brain gene expression data were obtained from the AHBA dataset^2^, which was derived from six human postmortem donors. The original expression data of more than 20,000 genes at 3,702 spatially distinct brain tissue samples were processed using a newly proposed pipeline (Arnatkeviciute et al., 2019). The dataset from AHBA was processed following recommendations from previous studies to ensure consistency and reproducibility of results. Partial Least Squares (PLS) regression was employed to investigate the regional dFCD maps in relation to the transcriptional activity of all 10,027 genes.

### Gene enrichment analysis

Gene enrichment analysis was performed utilizing the DAVID Functional Annotation Bioinformatics Microarray Analysis platform, accessible at https://metascape. org/gp/index.html#/main/step1. Gene Ontology (GO) was employed to assess biological functions, covering biological processes (BP), cellular components (CC), and molecular functions (MF). Pathway databases, particularly the Kyoto Encyclopedia of Genes and Genomes (KEGG) pathway, were utilized to evaluate pertinent biological pathways. Enrichment analysis plots were created with SRplot available at http://www.bioinformatics.com.cn/.

### Specific expression analysis

Cell type-specific expression analysis, brain region-specific expression analysis, and temporal-specific expression analyses were performed at doughertytools.wustl.edu/CSEAtool.html to identify the distinct cell types, brain regions, and developmental stages showing an overrepresentation of these genes. A specificity index probability (pSI) was utilized with two thresholds (0.01 and 0.001) to evaluate the enrichment of genes in specific terms relative to others.

### Protein-protein interaction analysis

Protein-protein interaction (PPI) analysis was conducted using STRING v11.0^3^ to explore whether genes linked to brain functional alterations could form a PPI network. PPI pairs with a confidence interaction score > 0.9 were extracted for analysis. Hub genes were identified as those within the top 15% of degree values, representing the number of edges connected to a gene. Furthermore, the Human Brain Transcriptome database^4^ was utilized to delineate the spatial-temporal expression trajectory of hub genes exhibiting the highest degree values.

**FIGURE 1:**
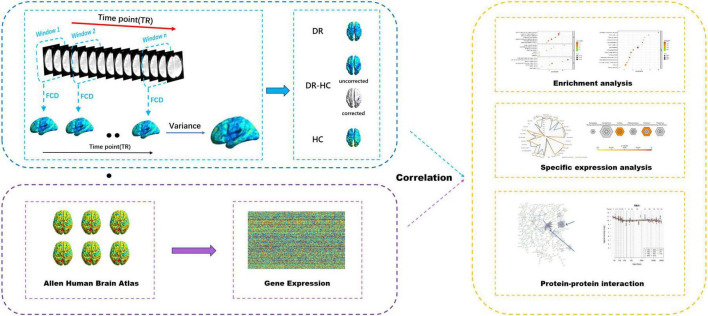
Overview of the analysis pipeline. Sliding window method was utilized to compute dFCD, the dFCD in the DR and HC groups were obtained by one-sample *t*-tests, and the dFCD differences between the two groups were compared by two-sample *t*-tests. Then the association between dFCD differences and gene expression from the AHBA was explored. Afterward, enrichment analysis, specific expression analysis and protein-protein interaction analysis were performed on this basis. dFCD, dynamic functional connectivity density; DR, diabetic retinopathy; HC, health control; AHBA, Allen Human Brain Atlas.

An overview of the analysis pipeline is illustrated in Figure 1.

### Statistical analysis

The demographic and clinical characteristics of the groups were analyzed using SPSS version 20.0 software (SPSS Inc., Chicago, IL, United States), employing the Chi-square test and independent-samples *t*-test, with a *p*-value of less than 0.05 considered indicative of a significant statistical difference. Intragroup patterns of dFCD maps were assessed through one-sample *t*-tests using SPM8 software.

Furthermore, two-sample *t*-tests were conducted to investigate group disparities in the dFCD maps (50TRs), employing the Gaussian random field (GRF) method for correcting multiple comparisons. Age and sex were regressed as covariates, and the analysis was performed with SPM8 software at a voxel level of *p* < 0.001 with GRF correction and a cluster level of *p* < 0.05.

Additionally, partial least squares (PLS) regression was utilized to explore the relationship between regional changes in dFCD and transcriptional activity for all 10,027 genes.

## Results

### Demographic and clinical characteristics

Significant differences were observed in best-corrected visual acuity (*P* < 0.001) between the two groups. However, there were no significant differences in sex, age, or body mass index between the two groups. Further details are provided in Table 1.

**TABLE 1:** Demographics and visual measurements between two groups.

Condition	DR group	HC group	t/χ^2^	P
Gender (male/female)	26/21	24/22	0.093	0.76
Age (years)	54.84 ± 3.22	55.36 ± 4.48	–0.462	0.65
Duration (months)	11.54 ± 4.37	–	–	–
Education	11.88 ± 2.05	11.68 ± 2.60	0.296	0.77
BCVA-OD	0.66 ± 0.15	1.11 ± 0.12	–11.523	<0.001*
BCVA-OS	0.64 ± 0.14	1.05 ± 0.18	–8.967	<0.001*
MoCA	25.37 ± 0.58	25.20 ± 0.74	0.927	0.358

BCVA, best corrected visual acuity; OD, oculus dexter; OS, oculus sinister; MoCA, Montreal Cognitive Assessment.

*Means a significant difference.

### Differences in dFCD variability between DR and HC

**FIGURE 2:**
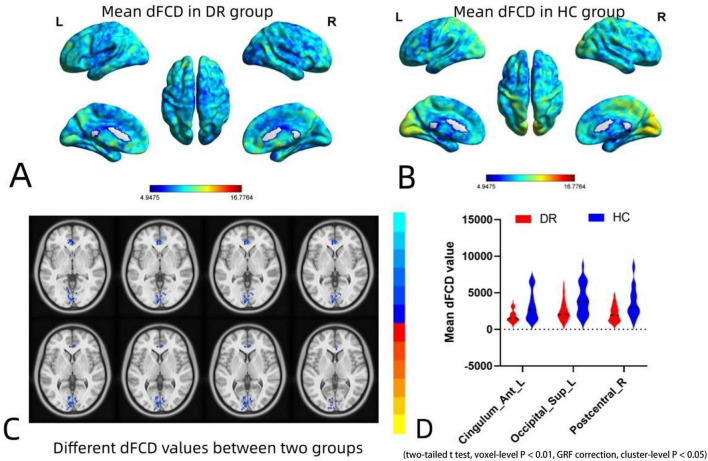
Spatial distribution maps of dFCD variability in the DR and HC groups and intergroup differences in dFCD values between the two groups. **(A,B)** Spatial distribution maps of dFCD variability in the DR and HC groups. The dFCD variability are normalized by subtracting the mean and dividing it by the SD of the global values and averaged across participants within each group. **(C,D)** Different dFCD values between two groups. Compared with HCs, DR group showed significantly decreased dFCD values in the left anterior cingulum, left superior occipital gyrus and right postcentral gyrus (two-sample *t*-tests with a two-tailed approach, voxel-level significance set at *P* < 0.01, Gaussian random field correction, and cluster-level significance at *P* < 0.05). dFCD, dynamic functional connectivity density; DR, diabetic retinopathy; HC, health control; GRF, gaussian random field.

The spatial distribution of dFCD variability in the DR group and the HC group is shown in Figures 2A,B. The results of the two-sample *t*-tests indicate that, compared to the HC group, the DR group exhibited significantly decreased dFCD values in the left anterior cingulum, left superior occipital gyrus and right postcentral gyrus (two tailed, voxel level of *p* < 0.001 with GRF correction and a cluster level of *p* < 0.05) (Figures 2C,D; Table 2).

**TABLE 2:** Group differences in dFCD variability between DR patients and HCs.

Brain regions	MNI coordinates			Peak *t*-values	Cluster size
	**x**	**y**	**z**		
**DR < HC**					
Cingulum_Ant_L	–9	36	–6	–4.3492	67
Occipital_Sup_L	–12	–99	9	–4.5470	82
Postcentral_R	33	–30	51	–4.8415	78

dFCD, dynamic functional connectivity density; MNI, Montreal Neurologic Institute; DR, diabetic retinopathy; HC, health control; R, right; L, left; Ant, anterior; Sup, superior.

### Gene enrichment results

**FIGURE 3:**
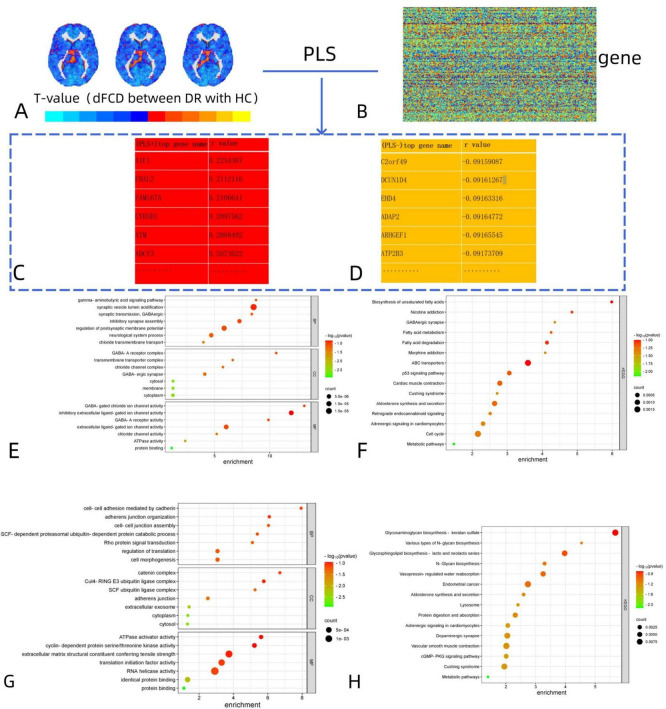
Functional enrichment of the genes associated with brain functional alterations in DR. **(A,B)** The association between dFCD differences and gene expression from the AHBA was analyzed. **(C,D)** The names of the most positively and negatively correlated genes were obtained on this basis. **(E,F)** The positive genes related to dFCD: GO analysis for BP, CC, and MF; Enriched KEGG pathways for dFCD variations. **(G,H)** The negative genes related to dFCD: GO analysis for BP, CC, and MF; Enriched KEGG pathways for dFCD variations. DR, diabetic retinopathy; dFCD, dynamic functional connectivity density; AHBA, Allen Human Brain Atlas; GO, gene ontology; BP, biological process; CC, cellular component; MF, molecular function; KEGG, Kyoto Encyclopedia of Genes and Genomes.

To elucidate the biological functions and pathways associated with brain functional alterations, the *t*-values of dFCD maps (Figure 3A) between two groups and gene (Figure 3B) was analyzed. we conducted functional enrichment analyses. For the positive genes related to dFCD, there were a total of 1,318 genes (Figure 3C). Gene Ontology (GO) analysis revealed 7 biological processes (BP), including gamma-aminobutyric acid signaling pathway, synaptic vesical lumen acidification, synaptic transmission GABAergic, inhibitory synapse assembly, regulation of postsynaptic membrane potential, neurological system process, and chloride transmembrane transport. Additionally, 7 cellular components (CC) were identified, such as GABA-A receptor complex, transmembrane transporter complex, chloride channel complex, GABA-ergic synapse, cytosol, membrane, and cytoplasm. Moreover, 7 molecular functions (MF) were annotated, including GABA-gated chloride ion channel activity, inhibitory extracellular ligand-gated ion channel activity, GABA-A receptor activity, extracellular ligand-gated ion channel activity, chloride channel activity, ATPase activity, and protein binding (Figure 3E). In terms of KEGG pathways, 15 pathways were enriched, including Biosynthesis of unsaturated fatty acids, Nicotine addiction, GABAergic synapse and Fatty acid metabolism, etc. (Figure 3F).

For the negative genes related to dFCD, there were also 1,318 genes identified (Figure 3D). GO analysis revealed 7 biological processes, such as cell-cell adhesion mediated by cadherin, adherens junction organization, cell-cell junction assembly, SCF-dependent proteasomal ubiquitin-dependent protein catabolic process, Rho protein signal transduction, regulation of translation, and cell morphogenesis. Additionally, 7 cellular components and 7 molecular functions were identified (Figure 3G), along with 15 enriched KEGG pathways, including Glycosaminoglycan biosynthesis-keratan sulfate, Various types of N-glycan biosynthesis, and Glycosphingolipid biosynthesis-lacto and neolacto series, etc. (Figure 3H).

### Specific expression analysis

**FIGURE 4:**
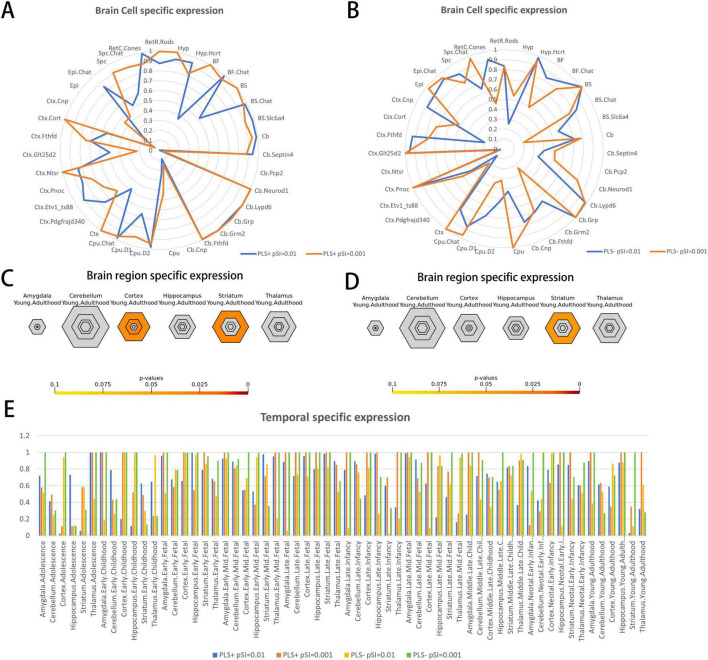
Specific expression of the genes associated with brain functional alterations in DR. **(A,B)** Brain cell type-specific expression. **(A)** Represent the results of brain cell-specific expression of PLS + genes and **(B)** represent the results of brain cell-specific expression of PLS- genes. The blue color in the radar plot represents the result at pSI = 0.01 and the orange color represents the result at pSI = 0.001. **(C,D)** Brain region-specific expression. **(C)** Illustrates the results of brain region-specific expression analysis of PLS + genes and **(D)** illustrates the results of brain region-specific expression analysis of PLS- genes. The results showed that PLS + genes (positive genes) were specifically expressed in the Cortex and Striatum, while PLS- genes (negative genes) were specifically expressed in the Striatum. The sizes of the hexagons denote brain region specificity across different specificity index probability (pSI) statistic thresholds ranging from 0.05 to 0.0001 (0.05, 0.01, 0.001, 0.0001). The outer hexagons correspond to the least specific test for a brain region (pSI threshold = 0.05), whereas the innermost hexagon reflects the most specific test for a brain region (pSI threshold = 0.0001). **(E)** Temporal-specific expression. As shown in the figure legend, the four colors represent the results of temporal specificity analysis of the two groups of genes at pSI = 0.01 and pSI = 0.001, respectively. DR, diabetic retinopathy; dFCD, dynamic functional connectivity density; PLS, partial least squares; pSI specificity index probability.

For the 1,318 positive genes associated with dFCD, cell-specific expression analysis revealed their distinct expression across various cell types, notably in Cb.Pcp2 (Figure 4A; Table 3). Moreover, brain-specific expression analysis highlighted their specific expression patterns in different brain regions, particularly in the Cortex and Striatum (Figure 4C; Table 4). Temporal-specific expression analysis further elucidated their differential expression across developmental stages and brain regions, however, no significant differential expression was found in this study (Figure 4E; Table 5).

**TABLE 3:** Brain cell type specific expression results.

Brain cell types and *P*-values	PLS + pSI = 0.01	PLS + pSI = 0.001	PLS- pSI = 0.01	PLS- pSI = 0.001
RetR.Rods	0.872	0.992	0.834	0.826
Hyp	0.927	1	0.257	0.543
Hyp.Hcrt	0.938	0.823	0.98	0.946
BF	0.366	1	0.833	0.487
BF.Chat	0.988	0.948	0.893	0.802
BS	0.515	1	1	1
BS.Chat	0.977	0.96	0.828	0.747
BS.Slc6a4	0.982	0.907	0.575	0.208
Cb	0.983	0.94	0.72	0.779
Cb.Septin4	0.937	0.878	0.402	0.512
Cb.Pcp2	0.002	0.039	0.353	0.523
Cb.Neurod1	0.997	1	0.512	0.802
Cb.Lypd6	0.999	1	0.982	0.976
Cb.Grp	0.984	1	0.998	1
Cb.Grm2	0.999	1	0.652	0.239
Cb.Fthfd	0.992	0.997	0.709	0.697
Cb.Cnp	0.091	0.158	0.767	0.812
Cpu	0.236	0.438	0.43	1
Cpu.D2	0.978	0.946	0.537	0.32
Cpu.D1	0.663	0.903	0.701	0.358
Cpu.Chat	0.977	0.961	0.868	0.965
Ctx	0.479	1.000	0.753	1
Ctx.Pdgfrajd340	0.755	0.590	0.1	0.209
Ctx.Etv1_ts88	0.910	0.670	0.405	0.501
Ctx.Pnoc	0.839	0.738	0.952	1
Ctx.Ntsr	0.839	1.000	0.041	0.061
Ctx.Glt25d2	0.501	0.227	0.958	1
Ctx.Fthfd	0.698	0.746	0.932	0.771
Ctx.Cort	0.551	1.000	0.209	0.717
Ctx.Cnp	0.074	0.053	0.881	0.521
Epi	0.208	0.446	0.889	0.974
Epi.Chat	0.848	0.446	0.929	0.948
Spc	0.524	0.908	0.877	0.774
Spc.Chat	0.609	0.890	0.646	0.971
RetC.Cones	0.987	0.885	0.913	0.465

PLS, partial least squares; pSI specificity index probability.

**TABLE 4:** Brain region specific expression results.

Brain regions and development and *p*-values	PLS + pSI = 0.01	PLS + pSI = 0.001	PLS- pSI = 0.01	PLS- pSI = 0.001
Amygdala.young.adulthood	0.396	1	0.154	1
Cerebellum.young.adulthood	0.582	0.985	0.315	0.594
Cortex.young.adulthood	0.005	0.585	0.922	0.868
Hippocampus.young.adulthood	0.586	0.62	0.981	0.964
Striatum.young.adulthood	0.392	0.448	0.067	0.942
Thalamus.young.adulthood	0.637	0.896	0.363	0.9

PLS, partial least squares; pSI specificity index probability.

**TABLE 5:** Temporal specific expression results.

Brain regions and development and *p*-values	PLS + pSI = 0.01	PLS + pSI = 0.001	PLS- pSI = 0.01	PLS- pSI = 0.001
Amygdala.adolescence	0.72	0.58	0.516	1
Cerebellum.adolescence	0.412	0.491	0.25	0.302
Cortex.adolescence	0.027	0.111	0.939	1
Hippocampus.adolescence	0.73	0.111	0.122	0.113
Striatum.adolescence	0.058	0.585	0.583	0.312
Thalamus.adolescence	0.998	1	0.441	1
Amygdala.early.childhood	1	1	0.182	1
Cerebellum.early.childhood	0.789	0.43	0.259	0.438
Cortex.early.childhood	0.199	1	1	1
Hippocampus.early.childhood	0.117	0.52	1	1
Striatum.early.childhood	0.626	0.486	0.298	0.135
Thalamus.early.childhood	0.647	0.234	0.968	0.237
Amygdala.early.fetal	0.959	1	0.512	1
Cerebellum.early.fetal	0.677	0.585	0.79	0.788
Cortex.early.fetal	0.657	1	0.992	1
Hippocampus.early.fetal	0.997	0.547	0.971	1
Striatum.early.fetal	0.788	1	0.861	0.954
Thalamus.early.fetal	0.683	0.653	0.478	0.899
Amygdala.early.mid.fetal	0.925	1	0.929	1
Cerebellum.early.mid.fetal	0.891	0.806	0.842	0.922
Cortex.early.mid.fetal	0.544	0.551	0.694	1
Hippocampus.early.mid.fetal	0.53	0.373	0.945	1
Striatum.Early.Mid.Fetal	0.977	0.718	0.858	0.355
Thalamus.early.mid.fetal	0.952	1	0.203	1
Amygdala.late.fetal	0.888	1	0.058	1
Cerebellum.late.fetal	0.718	1	0.731	1
Cortex.late.fetal	0.954	1	0.71	1
Hippocampus.late.fetal	0.798	1	0.807	1
Striatum.late.fetal	0.983	1	0.821	1
Thalamus.late.fetal	0.895	0.849	0.525	0.656
Amygdala.late.infancy	0.791	1	0.091	1
Cerebellum.late.infancy	0.896	0.857	0.759	0.443
Cortex.late.infancy	0.485	1	0.815	1
Hippocampus.late.infancy	0.985	1	0.261	0.704
Striatum.late.infancy	0.601	0.699	0.024	0.33
Thalamus.late.infancy	0.342	1	0.207	1
Amygdala.late.mid.fetal	0.99	1	0.945	1
Cerebellum.late.mid.fetal	0.913	0.685	0.527	0.876
Cortex.late.mid.fetal	0.626	1	0.091	1
Hippocampus.late.mid.fetal	0.22	0.835	0.963	0.839
Striatum.late.mid.fetal	0.462	0.769	0.62	1
Thalamus.late.mid.fetal	0.161	0.269	0.938	0.989
Amygdala.middle.late.childhood	0.251	1	0.839	1
Cerebellum.middle.late.childhood	0.718	1	0.433	0.906
Cortex.middle.late.childhood	0.742	0.699	0.341	0.704
Hippocampus.middle.late.childhood	0.647	0.551	0.656	1
Striatum.middle.late.childhood	0.817	0.835	0.725	0.839
Thalamus.middle.late.childhood	0.367	0.903	0.985	0.906
Amygdala.neotal.early.infancy	0.835	0.125	0.534	1
Cerebellum.neotal.early.infancy	0.423	0.293	0.441	1
Cortex.neotal.early.infancy	0.79	0.632	0.987	1
Hippocampus.neotal.early.infancy	0.855	1	0.117	1
Striatum.neotal.early.infancy	0.85	1	0.439	0.704
Thalamus.neotal.early.infancy	0.608	0.603	0.51	0.877
Amygdala.young.adulthood	0.896	1	0.384	1
Cerebellum.young.adulthood	0.614	0.634	0.526	0.272
Cortex.Young.Adulthood	0.589	0.349	0.863	0.723
Hippocampus.Young.Adulthood	0.876	1	0.881	1
Striatum.Young.Adulthood	0.034	0.349	0.113	1
Thalamus.Young.Adulthood	0.32	1	0.611	0.281

PLS, partial least squares; pSI specificity index probability.

In contrast, cell-specific expression analysis of the 1,318 negative genes linked to dFCD did not reveal significant expression (Figure 4B; Table 3). Brain-specific expression analysis showcased their distinct expression in different brain regions, notably in the Striatum (Figure 4D; Table 4). Temporal-specific expression analysis delineated their varied expression across developmental stages and brain regions, similar to the positive genes, we did not find significant differential expression (Figure 4E; Table 5).

### PPI network and hub genes

**FIGURE 5:**
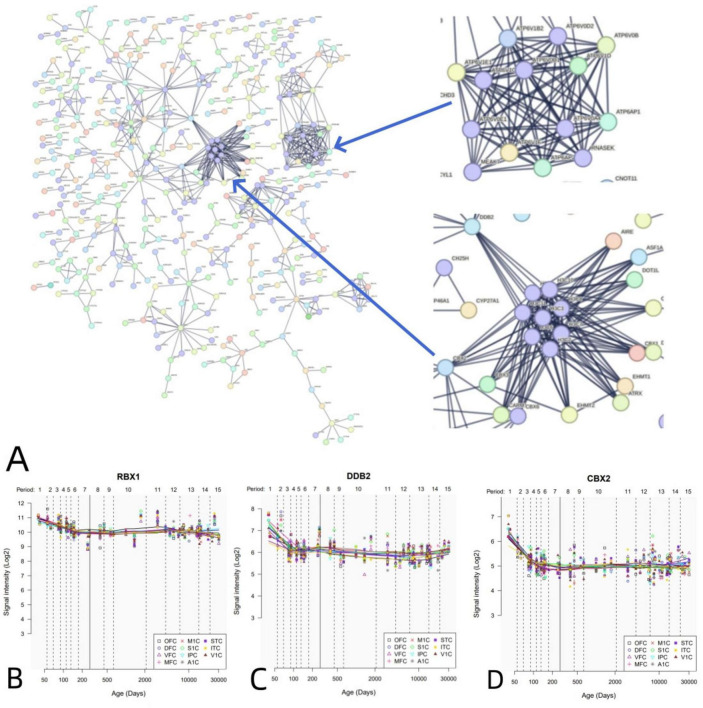
PPI network and hub genes. **(A)** A PPI network with 1,318 positive genes associated with dFCD. **(B-D)** Spatial and temporal expression curves of three hub genes with the highest degree values (i.e., RBX1, DDB2, and CBX2).

PPI analysis uncovered that 1,318 positive genes linked to dFCD formed an interlinked PPI network (Figure 5A). Additionally, we elucidated the spatial-temporal expression patterns of three key genes with the highest degree values: RBX1 (Figure 5B), DDB2 (Figure 5C), and CBX2 (Figure 5D).

**FIGURE 6:**
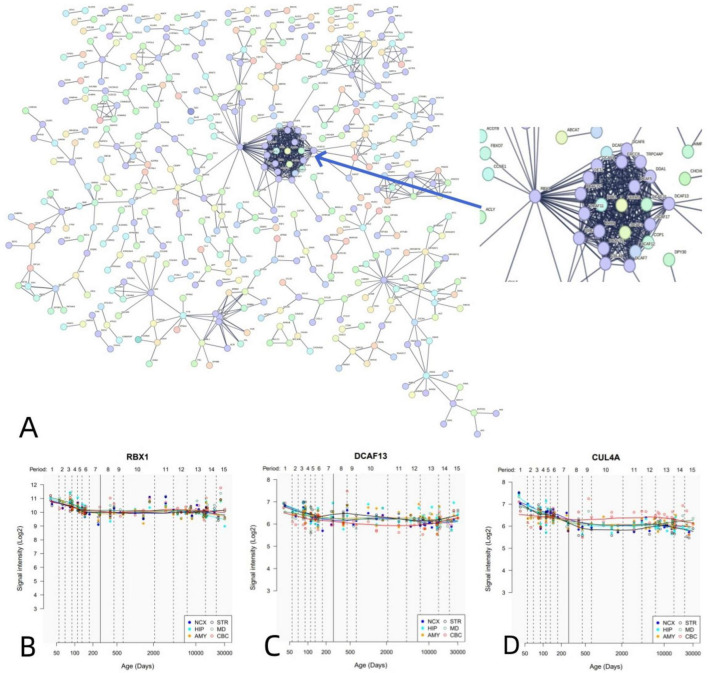
PPI network and hub genes. **(A)** A PPI network with 1,318 negative genes associated with dFCD. **(B-D)** Spatial and temporal expression curves of three hub genes with the highest degree values (i.e., RBX1, DCAF13, and CUL4A).

Similarly, PPI analysis revealed that 1,318 negative genes associated with dFCD constructed an interconnected PPI network (Figure 6A). We further outlined the spatial-temporal expression trajectories of three pivotal genes with the highest degree values: RBX1 (Figure 6B), DCAF13 (Figure 6C), and CUL4A (Figure 6D).

## Discussion

This study represents an innovative approach integrating functional magnetic resonance imaging with transcriptome-neuroimaging spatial correlation to explore the genetic underpinnings of dFCD variability in diabetic retinopathy (DR). Our investigation revealed a significant reduction in dFCD variability within the left anterior cingulum, left superior occipital gyrus, and right postcentral gyrus in the DR group compared to the healthy control (HC) group. Transcriptome-neuroimaging correlation analyses identified two groups of DR-related genes (PLS1 + and PLS1-) that exhibited substantial enrichment in biological functions and pathways related to ion channels, synaptic function, neuronal systems, and cellular junctions. Specific expression analyses indicated the unique expression of these genes in Purkinje neurons, the cortex, and striatum brain regions, with no developmental period-specific expression observed. Additionally, genes linked to altered brain function were capable of forming a protein-protein interaction (PPI) network supported by hub genes. These insights suggest that the disrupted brain function in DR patients arises from intricate interactions among multiple genes with diverse functional characteristics, offering novel perspectives on the molecular mechanisms linked to dFCD alterations in DR patients.

Significantly reduced dFCD variability was observed in the left anterior cingulum, left superior occipital gyrus, and right postcentral gyrus in the diabetic retinopathy (DR) group compared to the healthy control (HC) group. The anterior cingulate gyrus, a component of the salience network, plays a role in higher functions like attention allocation (Pardo et al., 1990), reward anticipation, decision making, and emotion (Jackson et al., 2006). A study by Liao et al showed significantly reduced ReHo values in the right anterior cingulate gyrus in patients with DR and hypothesized that this reflected dysfunction of the anterior cingulate gyrus (Liao X.-L et al., 2019). Similar to their results, we found significantly reduced dFCD variability in the left anterior cingulate gyrus in DR patients, which may imply anterior cingulate gyrus dysfunction and lead to corresponding cognitive impairment in DR patients. The superior occipital gyrus is located in the occipital lobe and is mainly responsible for the processing of visual information. It has been found that functional connectivity from the bilateral lingual gyrus to the left superior occipital gyrus was reduced in the DR group compared to healthy controls (Huang et al., 2021b), which suggests disrupted connectivity of the visual network and the dorsal visual pathway, and may reflect impaired top-down modulation and visuospatial function in patients with DR. In another study, gray matter density (GMD) in the right occipital lobe of DR patients was significantly lower than that of healthy controls (Wessels et al., 2006). In addition, there is a significant decrease in intra-network functional connectivity (FC) of the visual network (VN) in DR patients (Huang et al., 2020). Combining these studies, we suggest that the reduced dFCD variability in the left superior occipital gyrus may reflect impaired visual function in DR patients. The postcentral gyrus is located in sensorimotor network (SMN) (Chenji et al., 2016), which plays an important role in motion control. One study found reduced local clustering in the postcentral gyrus in DR patients, which correlates with motor speed (van Duinkerken et al., 2016). Postcentral gyrus is closely associated with PVA-related (primary visual areas) spontaneous brain activity (Wang et al., 2008), and their connection affects the processing of spatial visual information (Ringach et al., 1996). We hypothesize that reduced dFCD variability in the postcentral gyrus of DR patients may cause abnormalities in motor control, such as abnormal eye movements.

Transcription-neuroimaging spatial association analysis is an effective method to study the genetic mechanisms underlying neuroimaging phenotypes in the patient’s brain (Zhang et al., 2022), and can identify genes associated with neuroimaging phenotypes (Fornito et al., 2019). Using transcriptional-neuroimaging spatial association analysis, we identified two groups of genes, PLS + and PLS-, which correlate with changes in dFCD in DR patients. Enrichment analyses showed that these genes were mainly enriched for biological functions and pathways such as ion channels, synapses and cellular junctions. It was shown that ion channels are closely related to neuronal growth and differentiation, membrane potential generation, signal transduction and neurotransmitter release (Moody and Bosma, 2005; Smith and Walsh, 2020; Kumar et al., 2016; Hull and Isom, 2018). Alterations in the expression levels of somatostatin (SRIF) or specific SRIF receptors affect the morphology of retinal cell types (i.e., optic rod cells) and may influence transmitter release in photoreceptors by modulating voltage-gated K + and Ca2 + currents, making SRIF a potentially promising drug SRIF for the treatment of DR (Casini et al., 2005). 11-cis-retinal intervention improves aberrant outer retinal ion channel closure, thereby alleviate diabetes-induced outer retinal layer dysfunction (Berkowitz et al., 2012). This implies that ion channels may be an important therapeutic target for DR. Inter-neuronal communication is largely dependent on synaptic function (Pereda, 2014), and in hyperglycemia, both neurochemical (synaptic) and electrical (gap junction) modes of communication between retinal cells are affected (Eleftheriou et al., 2020). Diabetes causes a cumulative loss of neural dendrites and synapses in the inner retina, which results in significant thinning of the inner plexiform layer (IPL) (Barber et al., 1998). Hyperphosphorylated tau proteins contribute to diabetic retinal neurodegeneration by disrupting synaptic and mitochondrial function in retinal ganglion cells (RGCs) (Zhu et al., 2018). This suggests the importance of synaptic dysfunction in the pathophysiological process of DR. In DR, the blood-retinal barrier (BRB) is disrupted (Xu et al., 2011). Inflammatory cytokine levels are elevated in the vitreous fluid of patients with DR (Daruich et al., 2018), whereas pro-inflammatory cytokines have been found to cause down-regulation of tight junctions in bovine retinal endothelial cells through the NF-κB pathway, and, increase retinal endothelial cell permeability (Aveleira et al., 2010). In the mouse retina, hyperglycemic memory (HGM) induces sustained oxidative stress, mitochondrial membrane potential collapse and fission, adhesion junction disassembly and subsequent vascular leakage after normalization of blood glucose (Lee et al., 2022). The association between disruption of the blood-retinal barrier (BRB) and the down-regulation or disintegration of cellular connections is evident. Conversely, the overexpression of endothelial CYP2J2 preserves the distribution of endothelial tight junctions and adherens junctions in an ANXA1-dependent manner, thus safeguarding the integrity of the BRB and shielding retinal ganglion cells from loss following ischemia-reperfusion injury (Zhao et al., 2022).

The PLS + genes exhibited specific expression mainly in the cortex and striatum, while the PLS- genes showed predominant expression in the striatum, indicating a focus of diabetic retinopathy (DR) effects on the cerebral cortex. Moreover, the PLS + gene demonstrated particular expression in Purkinje neurons (Cb.Pcp2) located in the cerebellar cortex (Hirano, 2018), pivotal for integrating cerebellar inputs and outputs. The regular firing activity of Purkinje neurons is maintained through precise ion channel control (Raman and Bean, 1999), and ion channel dysregulation leads to perturbations in the intrinsic membrane excitability of Purkinje neurons, causing abnormal firing in Purkinje neurons (Bushart and Shakkottai, 2019; Cook et al., 2021; Huang and Shakkottai, 2023), which corresponds to the enrichment of these genes in ion channels. We did not find significant specific expression of PLS- genes at the cellular level. Furthermore, time-specific expression analyses, using predefined thresholds, did not reveal specific expression of dFCD-related genes at distinct developmental stages.

PPI analyses have revealed that dFCD-related genes can form a PPI network supported by functionally significant hub genes crucial for various biological processes. For instance, the RBX1 gene encodes RBX1 (RING box protein 1), essential for developmental processes. RBX1 suppression triggers the DNA damage response (DDR), resulting in G2-M arrest, senescence, and apoptosis (Jia et al., 2011). Another example is DDB2 (damage-specific DNA-binding protein 2), a component of the group E gene associated with dyschromic dry dermatosis, participating in the initiation of molecular ubiquitination within the ubiquitin ligase complex involving DDB1 and CUL4A (cullin 4A) (Stoyanova et al., 2009; Yan et al., 2011). DDB2 directly interacts with progestin and adipoQ receptor 3 (PAQR3), facilitating PAQR3 ubiquitination, consequently enhancing PI3K/AKT axis signaling to ameliorate insulin resistance and mitigate retinopathy in diabetic mice (Xiao et al., 2022).

Several limitations are evident in this study. Firstly, the gene expression data originated from the brains of six postmortem donors, while the neuroimaging data were obtained from 93 living participants (Zeng et al., 2012; Hawrylycz et al., 2015). To address the differential sources, we focused on genes displaying more conserved expression profiles by setting a DS threshold, facilitating our transcriptome-neuroimaging spatial correlation analysis. Secondly, due to limited gene expression data in the right hemisphere and notable variations between cortical and subcortical regions, only tissue samples from the left cerebral cortex were included, potentially introducing bias. Thirdly, oversampling in spatial correlation analyses is a common concern, which may inflate the rate of false positives. Lastly, gene identification related to dynamic functional connectivity density (dFCD) was achieved indirectly through spatial correlation analysis of transcriptional neuroimaging, lacking direct experimental validation. Further exploration and validation through animal experiments are recommended for future studies.

## Conclusion

Our study identified altered dFCD variability in brain regions linked to visual and cognitive functions in DR patients. This was manifested by significantly lower dFCD variability in the left anterior cingulate gyrus, left supraoccipital gyrus, and right postcentral gyrus in DR group. Moreover, transcriptome-neuroimaging correlation analyses revealed a spatial association between these dFCD changes and the genes with unique functional profiles. These genes were enriched in biologically significant functions and pathways, specific to certain cells and brain areas. Our research offers novel understandings of the genetic mechanisms influencing dFCD alterations in DR. The findings are also important for the diagnosis of DR and the discovery of potential therapeutic targets for DR.

## Data Availability

The raw data supporting the conclusions of this article will be made available by the authors, without undue reservation.

## Footnotes

1
http://www.rfmri.org/dpabi

2
http://www.brainmap.org

3
https://string-db.org/

4
http://hbatlas.org/
